# Interleukins in community-acquired pneumonia: from biomarkers to precision medicine

**DOI:** 10.3389/fimmu.2026.1774731

**Published:** 2026-02-24

**Authors:** Xiaoying Zhang, Tongshuo Zhang, Ruihui Geng, Luqing Wei, Hui Liu, Hanyu Shi

**Affiliations:** 1 Department of Internal Medicine, Hospital of the First Mobile Corps of the Chinese People’s Armed Police Force, Dingzhou, Hebei, China; 2 Department of Medical Innovation Research, Chinese People's Liberation Army (PLA) General Hospital, Beijing, China; 3 Department of Clinical Laboratory and Pathology, Jiangsu Provincial Corps Hospital of People's Armed Police (PAP), Yangzhou, China; 4 Department of Pulmonary and Critical Care, Characteristic Medical Center of the Chinese People’s Armed Police Force, Tianjin, China; 5 Department of Weigongqiao Outpatient, Jingxi Medical Section of People's Liberation Army (PLA) General Hospital, Beijing, China

**Keywords:** biomarkers, community-acquired pneumonia, interleukins, multimodal application, precision medicine

## Abstract

Community-acquired pneumonia (CAP) is still a leading cause of death due to infection globally, yet precise severity assessment remains a significant clinical problem. More than any other group of cytokines, interleukins are central to the regulation of inflammation and shed light on this intricate pathology. In the present review we summarize the biological and clinical characteristics of some of the principal interleukins (ILs) in CAP, classified primarily according to their physiological activity as pro-inflammatory (IL-2, IL-6, IL-8 and IL-12), anti-inflammatory (IL-7, IL-10 and IL-37), dual-action (IL-4 and IL-17), and emerging factors (IL-3, IL-27 and IL-33). Additionally, recent multimodal approaches are discussed such as combining cytokines with organ dysfunction parameters (MR-proADM) or revealing host-response patterns to inform antibiotic and corticosteroid management. We propose that the field needs to transition to immunological endotyping, multi-omics (integrating genetics and lung microbiome), and artificial intelligence (AI) models based on dynamic patient data to achieve precision medicine in CAP management.

## 1 Introduction

Community-acquired pneumonia (CAP) remains a major cause of death worldwide. According to the Global Burden of Disease Study (GBD), lower respiratory infections, primarily CAP, accounted for approximately 2.2 to 2.5 million deaths globally in recent years, ranking as the leading infectious cause of death worldwide (1). This burden is substantially elevated in vulnerable populations, especially the elderly and immunocompromised individuals, whose immunosenescence and comorbidities negatively impact the effectiveness of therapy (2). The disease has a wide spectrum of clinical severity from mild infection to fatal respiratory failure; thus, accurate risk stratification is essential for optimal management. Previously, clinical severity scores like the Pneumonia Severity Index (PSI) and CURB-65 are considered the standard for prognostication. However, these tools mainly depend on static physiological indicators (e.g., age, confusion, urea nitrogen) and frequently neglect to assess the host’s real-time inflammatory condition or identify accelerated deterioration in the early stages of infection (2, 3). As crucial mediators of the immune response, interleukins (ILs) serve as dynamic biomarkers indicating illness severity. In contrast to static clinical scores, these cytokines provide insight into the “cytokine storm” or immunoparalysis linked to negative outcomes (4). This systematic review assesses the biological roles of principal interleukins and their efficacy in evaluating the severity of CAP. Furthermore, we explore the clinical limitations, and the potential for a multi-marker strategy and future directions to better manage the condition.

## 2 Biological profiles and prognostic utility of major interleukins

### 2.1 Pro-inflammatory interleukins

#### 2.1.1 IL-2

**Figure 1:**
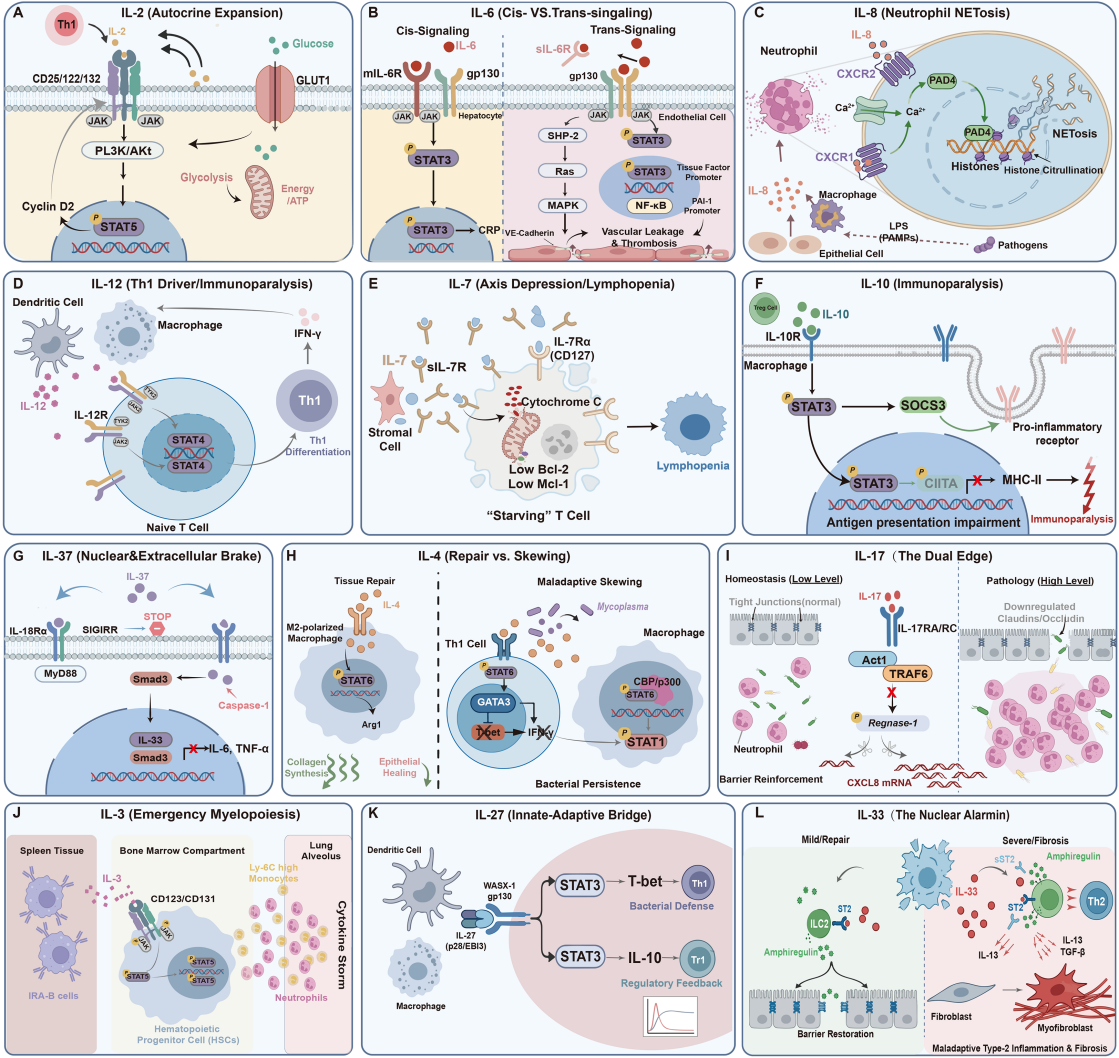
Molecular pathways of interleukins in CAP. **(A)** IL-2: IL-2 connects immune activation and metabolism. The PI3K/Akt axis promotes clonal proliferation by upregulating GLUT1 translocation and glycolysis, while STAT5 promotes cell cycle progression via Cyclin D2. **(B)** IL-6: Homeostatic *cis-signaling* (left) promotes CRP synthesis in hepatocytes via membrane-bound IL-6R. Conversely, pathological *trans-signaling* (right) engages gp130 on endothelial cells to activate the SHP-2/MAPK/STAT3 pathway, downregulating VE-cadherin (vascular leakage) and upregulating tissue factor (immunothrombosis). **(C)** IL-8: When IL-8 is bound to CXCR1/2, intracellular calcium activates peptidylarginine deiminase 4 (PAD4), which moves to the nucleus to citrullinate histones and resulting in cytotoxic Neutrophil Extracellular Traps (NETs). **(D)** IL-12: IL-12 activates the JAK2/TYK2-STAT4 pathway to drive Th1 differentiation, resulting in substantial IFN-γ production and macrophage activation. **(E)** IL-7: Increased soluble IL-7 receptors (sIL-7R) sequester IL-7, leading to diminished Bcl-2 and Mcl-1 expression in T cells, thereby inducing apoptosis-related lymphopenia. **(F)** IL-10: IL-10 turns on the STAT3/SOCS3 axis to suppress inflammation and represses CIITA transcription, resulting in decreased surface MHC-II and reduced antigen presentation. **(G)** IL-37: IL-37 inhibits macrophage cytokine release via two mechanisms: extracellular interaction with the IL-18R/SIGIRR complex and intracellular nuclear translocation involving Smad3. **(H)** IL-4: IL-4 promotes tissue repair via macrophage STAT6/Arg1 signaling (M2 phenotype) but causes bacterial persistence by inducing GATA3 and physically sequestering the co-activator CBP/p300, which blocks protective STAT1 signaling. **(I)** IL-17: Physiological IL-17 stabilizes epithelial barriers by stabilizing inflammatory mRNA and suppressing RNase Regnase-1, which support moderate neutrophil recruitment and barrier reinforcement. In contrast, excessive levels disrupt Claudin/Occludin tight junctions. **(J)** IL-3: Released by splenic Innate Response Activator (IRA) B cells, IL-3 induces “emergency myelopoiesis”. This process drives the rapid mobilization of Ly-6C monocytes and neutrophils to the lungs, thereby exacerbating the cytokine storm. **(K)** IL-27: IL-27 fine-tunes the immune response, driving protective Th1 immunity via STAT1 while inducing anti-inflammatory Tr1 cells via STAT3. **(L)** IL-33: Released from the nucleus of necrotic epithelial cells upon injury, transient increase of IL-33 initiates Amphiregulin-mediated epithelium healing, whereas persistent release stimulates IL-13/TGF-β synthesis, promoting the differentiation of fibroblasts into myofibroblasts and pulmonary fibrosis.

IL-2, generated exclusively by activated Th1 and CD8+ T cells, plays a crucial role in T-cell proliferation and immune regulation. It activates JAK1/3 and STAT5 via a trimeric receptor complex (CD25/CD122/CD132). After being phosphorylated, STAT5 moves to the nucleus where it promotes the transcription of cell cycle genes (Cyclin D2) and the IL-2Rα (CD25) gene itself (a positive feedback loop). This signaling is very important because it is paired with the activated PI3K/Akt/mTORC1 pathway, which makes GLUT1 more active and speeds up glycolysis (the Warburg effect), thus providing the biomass necessary for the proliferation of effector T-cells (**Figure 1A**). Simultaneously, low-level IL-2 signaling is required for regulatory T cells (Tregs), increasing B-cell lymphoma 2 (Bcl-2) expression and ensuring their survival and suppressive activity (5). In adult CAP, circulating IL-2 levels are markedly increased relative to healthy controls (Median: 5.67 vs. 2.13 pg/mL; p < 0.001), signifying active infection and corroborating its use as a diagnostic adjunct (6).

However, its utility varies greatly with age. In adults, levels correlate with severity scores (CURB-65, PSI). Its overall performance is moderate but a value> 10.40 pg/mL offers the highest specificity (97%) for death of all markers and independently predicts mortality (HR 1.016), outperforming IL-6 in differentiating high-risk status (6). In the elderly (≥65 years), however, its prognostic significance weakens, probably reflecting immunosenescence and impaired T-cell responses (7). Pediatric observations suggest a more nuanced picture: whereas bronchoalveolar lavage fluid (BALF) levels are locally raised, systemic levels may be decreased in severe cases, suggesting a possible protective impact via Treg activation in early life (8, 9).

#### 2.1.2 IL-6

M1-polarized macrophages and endothelial cells are the principal sources of IL-6 during the acute phase. They respond to recognizing pathogens and control the acute-phase reaction as a whole. Biologically, IL-6 exerts pleiotropic effects via two distinct signaling paradigms. First, homeostatic *cis*-signaling occurs when IL-6 binds membrane-bound IL-6R on hepatocytes and immune cells. This activates the JAK/STAT3 pathway to induce C-reactive protein (CRP) synthesis and, crucially, promotes resolution by stimulating the release of anti-inflammatory mediators like IL-1Ra and IL-10. Second, under inflammatory stress, the pathological *trans*-signaling pathway dominates. Here, the soluble IL-6R/gp130 complex activates the SHP-2/Ras/MAPK signaling pathway in cells lacking membrane receptors (e.g., endothelial cells, **Figure 1B**). While this mechanism is necessary for leukocyte recruitment, its excessive activation blocks VE-cadherin and strengthens the NF-κB loop, which leads to the cytokine storm and vascular leakage (10, 11). This eventually results in systemic inflammatory response syndrome (SIRS), acute respiratory distress syndrome (ARDS), and multiple organ dysfunction syndrome (MODS) (12). Crucially, as a primary stimulator of tissue factor and plasminogen activator inhibitor 1 (PAI-1), elevated IL-6 links inflammation and coagulation, thereby predisposing severe CAP patients to immunothrombosis and pulmonary embolism (13).

Clinically, the extent of this inflammatory response correlates significantly with patient outcomes. Regarding its diagnostic value, serum IL-6 levels in healthy baselines are modest (~5 pg/mL), whereas systemic values above 100 pg/mL indicate deteriorating oxygenation and radiographic deterioration (14). Moreover, significantly elevated amounts differentiate bacteremic CAP (Mean: 2,852 pg/mL) from viral or other causes (≤300 pg/mL). In addition, IL-6 has higher prognostic performance over PSI for predicting 30-day mortality (AUC 0.934) and offers an early diagnosis window 24-48h before the development of symptoms (7, 15). Longitudinal surveillance is necessary; sustained titers in older patients independently predict death (OR 5.39) for the indication of antibiotic escalation (7). Moreover, the efficacy of IL-6 receptor antagonists (e.g., Tocilizumab, Sarilumab) in decreasing mortality among critically ill patients (the RECOVERY and REMAP-CAP trials) has substantiated the *trans*-signaling pathway as a viable therapeutic target, representing a paradigm change in the management of severe viral CAP (10).

#### 2.1.3 IL-8

Within 72 hours after symptom onset, alveolar macrophages and epithelial cells produce IL-8, which serves as a chemoattractant for neutrophils. It stimulates the PI3K/Akt pathway by binding to the G-protein-coupled receptors CXCR1 and CXCR2. This controls not only chemotaxis but also respiratory burst and degranulation (**Figure 1C**) (16). Mechanistically, IL-8 binding triggers a rapid intracellular calcium influx. In cases of severe infection, this calcium surge directly triggers the nuclear translocation of peptidylarginine deiminase 4 (PAD4). PAD4 citrullinates histones, which makes chromatin less dense and releases Neutrophil Extracellular Traps (NETs) in an enormous way. These released DNA-histone complexes have direct cytotoxic effects on the alveolar epithelium, which leads to consolidation and immunothrombosis (17, 18).

Given its direct contribution to tissue injury, IL-8 is an effective risk classification tool, especially in pediatric and severe adult CAP. In a pediatric cohort, IL-8 levels were significantly higher in severe cases compared to non-severe cases (Median: 120 vs. 50 pg/mL; p < 0.001) and showed superior predictive capability for in-hospital mortality compared to CRP and procalcitonin (PCT) (AUC 0.877 vs. 0.75 and 0.80, respectively), establishing itself as an independent prognostic factor (19). In adults, IL-8 levels are also linked to PSI scores and the probability of hospitalization. Inpatients have much higher levels than outpatients (Median: 34,763 vs. 14,696 pg/mL; p = 0.001), and they are much more likely to need mechanical ventilation (HR 1.42) (20). Unlike the temporary rise in IL-6, the increase in IL-8 often persists for 3–5 days or longer in unresponsive patients (16), which is associated with longer-lasting lung damage and the development of ARDS (21).

#### 2.1.4 IL-12

IL-12 is usually produced by activated dendritic cells (DCs) and macrophages in response to microbial stimulation. It functions as a key regulator of type 1 immunity, regulating innate and adaptive immune responses (**Figure 1D**). By interacting with the IL-12R/β1/β2 complex on NK cells and naive T cells, IL-12 stimulates the JAK2/TYK2/STAT4 signaling pathway. This activation is essential for promoting the differentiation of Th1 cells and resulting in substantial production of interferon-γ (IFN-γ), which is important for activating macrophages to eliminate intracellular pathogens (e.g., Legionella, viruses) (22).

In the context of CAP, the role of IL-12 is dependent on both the stage of the disease and its underlying cause. During the initial hyper-inflammatory phase of viral pneumonia, such as Influenza A, increased levels of IL-12, along with IL-6, exacerbate the cytokine storm and subsequent tissue damage. Conversely, a diminished IL-12 response is frequently observed in cases of severe bacterial sepsis. This phenomenon, termed “immunoparalysis”, is characterized by monocyte exhaustion and the downregulation of HLA-DR (Major Histocompatibility Complex, Class II, DR Alpha), which correlates with an impaired ability to resolve secondary infections (23). In addition, reduced levels of IL-12p70 in septic patients have been linked to heightened mortality, thereby serving as a biomarker for immune suppression that may be responsive to immune-stimulating treatments rather than anti-inflammatory interventions (20).

### 2.2 Anti-inflammatory cytokines

#### 2.2.1 IL-7

The IL-7/IL-7R axis is necessary for the survival and proper growth of both naïve and memory T cells. Instead of hematopoietic sources, IL-7 is primarily secreted by stromal and epithelial cells in the bone marrow and lymphoid tissues, essential for maintaining the T-cell pool. When CD127 (IL-7Rα) delivers a signal, it activates STAT5, which raises the levels of anti-apoptotic molecules (Bcl-2, Mcl-1) to prevent cell death. In severe CAP, this axis shows significant “axis suppression,” which is marked by low levels of IL-7 and high levels of soluble IL-7R (sIL-7R, a decoy receptor, **Figure 1E**). This dysregulation directly leads to the absolute lymphopenia that is often seen in sepsis, making it more challenging for the immune system to clear viruses and increasing the probability of developing secondary infections (24, 25).

Clinically, this depletion is apparent as severe CAP patients suffer from significantly reduced IL-7 levels (Median: 6.8 vs. 12.4 pg/mL) alongside elevated sIL-7R levels. sIL-7R (>25.04 ng/mL) functions as an independent predictor of 30-day mortality (HR 3.42) (24). Consistent with this, external IL-7 administration could ameliorate sepsis-induced lymphopenia and increase CD4+/CD8+ T-cell counts (26). Moreover, decreased IL-7R expression in BALF is associated with an increased pathogen burden, supporting its function in local immune competence (27).

#### 2.2.2 IL-10

IL-10, mostly derived from Tregs, Th2 cells, and M2 macrophages, functions as a powerful immunoregulator. It interacts with the IL-10R1/R2 complex and activates the JAK1/TYK2/STAT3 axis. Phosphorylated STAT3 activates SOCS3 to inhibit pro-inflammatory signaling. More importantly, it blocks the transcription factor CIITA. This epigenetic silencing reduces the expression of surface HLA-DR on monocytes, which makes it harder for them to deliver antigens (**Figure 1F**) (28). Nevertheless, its role within severe CAP is paradoxical: high levels often signify a robust compensatory response to intense inflammation. This intense immunosuppressive state sets the stage for subsequent bacterial superinfections such as post-influenza aspergillosis and *S. aureus* pneumonia, which are major drivers of late mortality (29).

The IL-6/IL-10 ratio is an important indicator for evaluating this subtle balance between pro-inflammatory drive and compensatory anti-inflammatory resolution. Mechanistically, these two cytokines are closely linked via a negative feedback loop: circulating IL-6 acts as a regulatory factor that directly induces the transcription of IL-10 in T cells and macrophages (discussed in Section 2.1.2). In a functional immune system, a surge in IL-6 triggers a proportional rise in IL-10 to limit tissue damage. Therefore, a pathologically high IL-6/IL-10 ratio signifies not merely excessive inflammation, but a disruption of this homeostatic coupling—indicating that the compensatory anti-inflammatory response is insufficient to counteract the cytokine storm. Clinically, this imbalance is a potent predictor of adverse outcomes. For example, a ratio of 9.61 or more in children correlates with severe disease (PPV 93%) (30), whereas IL-10 levels >14.7 pg/mL after 48 hours differentiated nonsurvivors in adults more specifically than any other variable (96% specificity) (31). Genetic predisposition, along with circulating levels, also affects severity. The IL-10-1082G/A polymorphism is inherently associated with disease progression, with the G allele and GG genotype being markedly prevalent in severe and fatal cases (32). Thus, long-term elevation of IL-10 indicates not simply “anti-inflammation” but also immunoparalysis, a lethal status to monitor for complications occurring in hospitals.

#### 2.2.3 IL-37

Activated macrophages and epithelial cells produce IL-37, a broadly effective suppressor of innate immune responses. Mechanistically, it operates by a dual mechanism (**Figure 1G**): extracellularly, it binds to IL-18Rα and recruits SIGIRR (IL-1R8) to produce a “dead” decoy complex that can suppress NF-κB signaling; intracellularly, caspase-1-cleaved IL-37 translocates to the nucleus in the presence of Smad3 to epigenetically mute pro-inflammatory genes (33). Clinically, the serum level of IL-37 is inversely correlated with illness severity, duration of hospitalization, and mortality in CAP (34). Combining IL-37 with PSI provides a better prediction accuracy (AUC 0.911) than using risk scores alone. However, its impact is markedly pathogen-dependent. In viral pneumonia (e.g., Influenza), IL-37 is protective, attenuating excessive lung injury (34, 35). Conversely, in bacterial models (*S. pneumoniae*), IL-37-mediated immunosuppression impairs neutrophil recruitment and bacterial clearance, paradoxically worsening survival (36). Thus, evaluating IL-37 levels requires a classification of the pathogen within this dual role.

### 2.3 Dual-function cytokines

#### 2.3.1 IL-4

IL-4 is the main cytokine for Th2 cells, basophils, and type-2 innate lymphoid cells (ILC2). It has two roles that are controlled by IL-4Rα/STAT6 signaling. Its role in CAP varies significantly depending on the situation (37). In the context of recovery, such as convalescent viral or pediatric CAP, IL-4 offers protection by promoting the polarization of alveolar macrophages towards the reparative M2 phenotype, thereby enhancing Arginase-1 (Arg1) to generate polyamines for collagen synthesis and epithelial regeneration (38) (**Figure 1H**).

During acute bacterial or intracellular infections (*Mycoplasma, Chlamydia*), IL-4 induces a maladaptive Th2 skewing. This suppression starts in T-cells, where IL-4-activated STAT6 triggers GATA3, which transcriptionally silences T-bet. This stops the generation of IFN-γ, which is needed to activate macrophages. The problem is exacerbated downstream within macrophages: activated STAT6 physically locks away the limited transcriptional co-activators CBP/p300, hindering the bactericidal STAT1 signaling pathway even though there are still cytokines around (39). Clinically, high levels of IL-4 are linked to lobar consolidation and severity in Mycoplasma pneumonia, which is a sign of harmful immunologic deviation (40).

#### 2.3.2 IL-17

IL-17, mainly produced by Th17 and γδ T cells, facilitates mucosal defense (**Figure 1I**). Rather than act directly on neutrophils, it interacts with the IL-17RA/RC complex on epithelial and endothelial cells, bringing in the adaptor Act1 to recruit TRAF6 as well as phosphorylate and inactivate the RNase Regnase-1. This post-transcriptional mechanism stabilizes the mRNA of chemokines (e.g., CXCL8, IL-6), which allows rapid and efficient neutrophil mobilization (41). This process is crucial for strengthening the epithelial barrier and eliminating extracellular bacteria (*S. pneumoniae, A. baumannii*); knockout mice models demonstrate markedly decreased survival owing to impaired clearance (42, 43).

However, when IL-17 levels exceed a particular threshold (>50 pg/mL), it causes a “hyper-inflammatory” state. In this setting, it actively lowers the amounts of tight junction proteins like Claudins and Occludin, which weakens the epithelial barrier and allows excessive neutrophils to enter (44). The increase is frequently stimulated by mucosal-associated invariant T (MAIT) cells, which show skewed activation in severe pediatric CAP (45).

### 2.4 Emerging biomarkers

#### 2.4.1 IL-3

Traditionally regarded as a hematopoietic growth factor, IL-3 has recently been found as a significant regulator in sepsis. This cytokine is mostly released by a specific kind of B cells, called Innate Response Activator (IRA) B cells, during sepsis caused by pneumonia. When recognizing infectious substances, these cells migrate to the spleen and lungs. There, they release IL-3, which then travels via the bloodstream to the bone marrow. This signaling promotes “emergency myelopoiesis”, driving the fast proliferation and mobilization of Ly-6C monocytes and neutrophils, thereby exacerbating the systemic inflammatory response (**Figure 1J**) (46).

Clinically, IL-3 acts as a “double-edged sword” biomarker, with its effects dependent on the particular cause. In bacterial sepsis, high levels of IL-3 in the blood (>127.5 pg/mL) are linked to increased inflammation and independently predict death. In contrast, in severe viral pneumonia (e.g., SARS-CoV-2), IL-3 protects the lungs by recruiting plasmacytoid DCs for local antiviral defense. Consequently, low IL-3 levels in viral sepsis are associated with viral reactivation and poor prognosis (47, 48). Thus, IL-3 serves as a context-dependent biomarker: marked elevation warns of bacterial-induced hyper-inflammation, whereas depletion signals viral-associated immune failure.

#### 2.4.2 IL-27

As a member of the IL-12 family, IL-27 is mainly released by macrophages and DCs. It is an important immunoregulator that connects innate and adaptive immunity. The cytokine uses a “dual-signaling” signaling pathway through WSX-1/gp130 (**Figure 1K**). This stimulates STAT1 to promote Th1 differentiation (through T-bet induction) and enhance bacterial clearance. It also activates STAT3 to make Tr1 cells that make IL-10 and inhibit Th17 cell proliferation (49).

Its kinetic profile offers an additional benefit for clinical use. Unlike the transient spike observed in IL-6, IL-27 shows a more sustained rise that can persist for 72–96 hours and longer during bacterial infections (50), which gives a wider diagnostic window (51). Studies on pediatric and adult CAP demonstrate that IL-27 outperforms PCT in differentiating between bacterial and viral etiologies (AUC>0.85) (52). Besides diagnosis, admission levels are tightly linked to the level of bacteria burden and the severity of sepsis. IL-27 is an independent predictor of prolonged hospital stays and death in critically ill patients (51). These characteristics make IL-27 an appealing option for guiding antibiotic stewardship decisions.

#### 2.4.3 IL-33

IL-33 is usually kept in the nuclei of pulmonary epithelial cells, which is different from circulating cytokines that leukocytes release. It acts as an “alarmin” by being passively released upon cellular necrosis, delivering signals to ILC2s and Th2 cells through the ST2 (IL-1RL1) receptor and MyD88 pathway (**Figure 1L**). This distinct release mechanism makes IL-33 a direct biomarker for epithelial barrier integrity and lung tissue damage (46). When the system is stable or has only mild injury (like seasonal flu), IL-33 helps repair tissue. It accomplishes this by prompting ILC2s to secrete Amphiregulin (Areg), an epidermal growth factor essential for epithelial regeneration (47).

In cases of severe viral CAP (e.g., SARS-CoV-2), the situation alters. In this instance, persistent and excessive IL-33 release disrupts the reparative pathway, leading to maladaptive type-2 inflammation, airway hyper-responsiveness, and pulmonary fibrosis. Clinically, serum IL-33 levels correlate with the radiographic severity of viral pneumonia and assist in predicting development to ARDS (48). sST2 (the soluble decoy receptor) is generally a better predictor of outcomes than IL-33 itself. High sST2 levels are a strong independent predictor of 30-day mortality and complications from heart failure in hospitalized CAP patients (49).

## 3 Integrative clinical strategies

**Figure 2:**
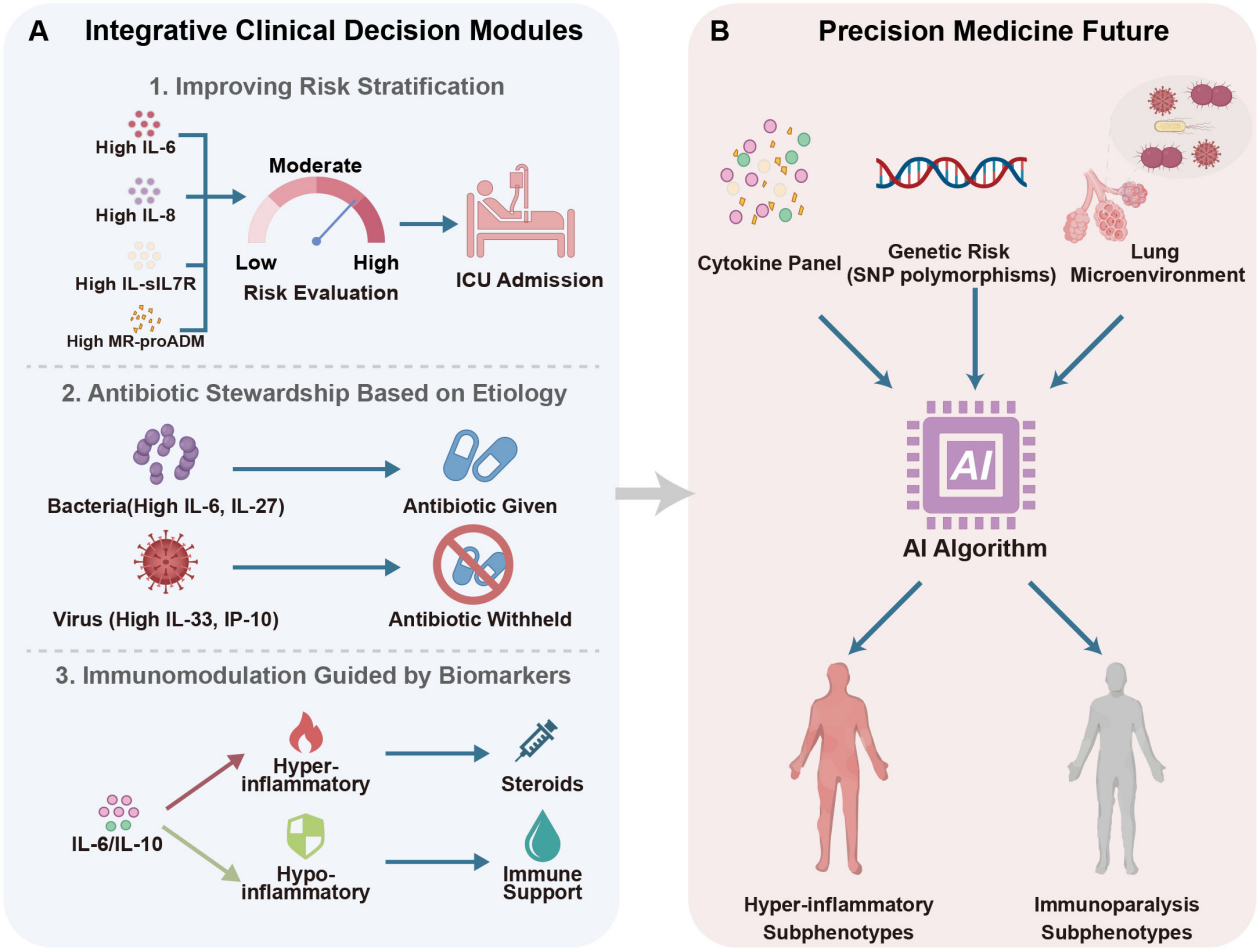
Integrative clinical decision modules and precision medicine framework. **(A)** Integrative Clinical Decision Modules. Module 1 enhances risk stratification by combining inflammatory cytokines with organ stress markers (MR-proADM). Module 2 guides antibiotic stewardship using etiology-specific signatures (IL-6/IL-27 for bacteria vs. IL-33/IP-10 for viruses). Module 3 directs immunomodulation by differentiating hyper-inflammatory (steroid-responsive) from immunoparalysis (immune support) phenotypes. **(B)** Precision Medicine Future. Integrating multi-omics (cytokines, genetics, lung microbiome) and AI modeling to identify latent subphenotypes for precise medicine.

To overcome the limitations of single-biomarker assays, clinical research is concentrating on multimodal integration (**Figure 2A**).

### 3.1 Improving risk stratification

Adding cytokines to physiological scores can create strong hybrid models. For instance, adding IL-6 and TNF-α to CURB-65 significantly improves the ability to predict death, often resulting in a positive Net Reclassification Improvement (NRI) that correctly moves “low-risk” patients into higher-risk groups (53, 54). Besides the standard use of IL-6, IL-8 levels especially correlate with the degree of alveolar damage and the need for mechanical ventilation, providing further detail to overall sepsis scores (20). Furthermore, indicators of adaptive immunity, such as sIL-7R, may identify individuals at high risk of late death due to severe lymphopenia (24). An even better strategy is to combine inflammatory triggers with signs of damage to the endothelium. Mid-regional pro-adrenomedullin (MR-proADM), which measures vascular permeability, performs well with the interleukins during the acute phase. This combinatorial panel (IL-6+MR-proADM) efficiently delineates the clinical progression and exhibits improved prediction accuracy (AUC 0.78–0.85) compared to single markers (54).

### 3.2 Antibiotic stewardship based on etiology

Not only for severity, but discerning viral from bacterial etiologies is important for antibiotic stewardship. While there is some overlap in individual markers, multi-marker panels can provide better discriminatory performance. In many cohorts, bacterial pneumonia is generally associated with elevated IL-6, IL-27 and PCT levels, while viral infections like influenza and COVID-19 have a distinct interferon-chemokine signature with increased IP-10 and IFN-γ. By combining these indicators—elevated IL-27/IL-6 for bacterial confirmation vs. elevated IP-10/IFN-γ for viral identification—clinicians may make more accurate judgements to refrain from prescribing antibiotics in viral CAP, hence reducing resistance rates (55).

### 3.3 Immunomodulation guided by biomarkers

ILs also have therapeutic potential for guiding immunomodulation treatment. The recent success of hydrocortisone in severe CAP (CAPE COD trial) and IL-6 receptor blockade in COVID-19 highlights the central role of immunomodulation (56). Nevertheless, unselective employment is still up for debate. To get the most benefit while causing the least harm, predictive enrichment may be needed. Identifying “hyper-inflammatory” phenotypes, which are likely to react to anti-inflammatory treatments, includes utilizing high levels of IL-6 or IL-6/IL-10 ratios (30). In contrast, indicators of immunoparalysis such as lower IL-7 levels or consistently elevated IL-10 concentrations, may help identify individuals who might benefit from immune-stimulatory treatments, such as recombinant IL-7, instead of immunosuppressive medicines (26). This change is the next step in managing CAP.

## 4 Limitations and future directions

Even though they have plenty of promise, applying ILs in clinical settings encounters biological and practical restrictions. Biologically, most ILs are not specific enough, since their levels increase in multiple situations including trauma and autoimmunity (57). Heterogeneity is also an important challenge. For example, IL-17 might be protective or detrimental depending on the pathogen and the host’s genes, which makes it hard to establish universal diagnostic cutoffs. On the operational side, the rapid dynamics of cytokines (IL-6 half-life is <6 hours) and the variances in testing methodologies (ELISA vs. CLIA) make it hard to compare facilities and employ them quickly in situations of emergency (58).

To bridge the gap from bench to bedside, future work must shift focus from validating single indicators to characterizing overall immunological states (**Figure 2B**). First, the scientific community needs to start using immunological endotyping. Following the ARDS research (59), future studies could use Latent Class Analysis (LCA) to stratify biomarker panels (like IL-6 and IL-8) into unique “hyper-inflammatory” subphenotypes. Moreover, this stratification should also integrate host genetics and microbiome profiles, as cytokine levels do not exist in a vacuum. For example, single-cell transcriptome investigations have shown that the lung microenvironment is a complex system. In this system, certain bacterial communities influence the development of immune cells, which directly affects the local cytokine response (60). Similarly, genetic variables in the host, such as IL-6 promoter polymorphisms (-174G/C), could alter the baseline inflammatory response and the likelihood of death (61). Integration of these data (local immune signs, genetic risk (SNPs), and microbial imbalances) would aid in clinicians making decisions on whether inflammation is more microbiome-driven (secondary to microbial shifts) or host-driven (due to poor regulation of immune function) (60).

And lastly, the shift to precision medicine requires the application of AI models, not only as a novel tool, but as a crucial analysis tool. Traditional linear scoring methods aren’t enough to handle the complexities of integrating high-dimensional data, which includes cytokines, genetic information, and time-series signs. Advanced machine learning methods, such as Random Forests and Long Short-Term Memory networks (LSTM), are essential for understanding non-linear interactions and identifying phenotypes that are hidden within noise (62). Furthermore, AI may forecast clinical deterioration hours before physiological collapse since it can capture the dynamic “progress” of inflammation rather than static data (63). This approach essentially focuses on precise theranostics in the management of CAP.

## 5 Conclusion

ILs play an important role in the pathogenesis of CAP, monitoring the transition from physiological defense to organ damage. Although traditional ILs (IL-6, IL-10) and novel candidates (IL-33, IL-27) provide important perspectives, the heterogeneity of the disease calls for an approach towards multimodal integration. Future advancements should be made via immunological endotyping, multi-omics signatures, and AI models to understand the immune landscape. Finally, it is important to turn this knowledge into precise theranostics to reduce mortality in this globally lethal syndrome.
